# Research on Machining Characteristics of C/SiC Composite Material by EDM

**DOI:** 10.3390/mi16121423

**Published:** 2025-12-18

**Authors:** Peng Yu, Ziyang Yu, Lize Wang, Yongcheng Gao, Qiang Li, Yiquan Li

**Affiliations:** Ministry of Education Key Laboratory for Cross-Scale Micro and Nano Manufacturing, Changchun University of Science and Technology, Changchun 130022, China; 17678358766@163.com (Z.Y.); 15665938736@163.com (L.W.); 15704301002@163.com (Y.G.); lqxuesheng@163.com (Q.L.)

**Keywords:** machining characteristics, electrical discharge machining, C/SiC composite material, micro hole

## Abstract

Carbon fiber reinforced silicon carbide (C/SiC) composite material exhibits exceptional properties, including high strength, high stiffness, low density, outstanding high-temperature performance, and corrosion resistance. Consequently, they are widely used in aerospace, defense, and automotive engineering. However, their anisotropic, high hardness, and brittle characteristics make them a typical difficult-to-machine material. This paper focuses on achieving high-quality micro hole machining of C/SiC composite material via electrical discharge machining. It systematically investigates electrical discharge machining characteristics and innovatively develops a hollow internal flow helical electrode reaming process. Experimental results reveal four typical chip morphologies: spherical, columnar, blocky, and molten. The study uncovers a multi-mechanism cutting process: the EDM ablation of the composite involves material melting and explosive vaporization, the intact extraction and fracture of carbon fibers, and the brittle fracture and spalling of the SiC matrix. Discharge energy correlates closely with surface roughness: higher energy removes more SiC, resulting in greater roughness, while lower energy concentrates on m fibers, yielding higher vaporization rates. C fiber orientation significantly impacts removal rates: processing time is shortest at θ = 90°, longest at θ = 0°, and increases as θ decreases. Typical defects such as delamination were observed between alternating 0° and 90° fiber bundles or at hole entrances. Cracks were also detected at the SiC matrix–C fiber interface. The proposed hole-enlargement process enhances chip removal efficiency through its helical structure and internal flushing, reduces abnormal discharges, mitigates micro hole taper, and thereby improves forming quality. This study provides practical references for the EDM of C/SiC composite material.

## 1. Introduction

Carbon fiber reinforced silicon carbide (C/SiC) composite material exhibits a unique combination of advantageous properties, including low density, high mechanical strength, excellent wear resistance, and superior chemical corrosion resistance [1,2,3]. Furthermore, the fiber reinforcement and toughening effect significantly enhance their fracture toughness, endowing them with broad application prospects in aerospace [4], energy, advanced structural [5], protection system [6], and defense applications [7,8]. However, as a typical ceramic matrix composite, C/SiC is notoriously difficult to machine, primarily due to its high hardness [9,10], heterogeneous fiber-woven structure, and the distinct mechanical properties of its constituent phases (carbon fibers and SiC matrix). Traditional mechanical machining methods often suffer from issues such as low processing accuracy, poor surface integrity, severe tool wear, and thermal damage induced by cutting heat, which greatly limit their practical application [11,12,13].

In recent years, non-traditional machining technologies have been increasingly employed for the processing of ceramic matrix composites, including high-pressure water jet machining, ultrasonic machining, laser processing, electric discharge machining (EDM), laser-assisted cutting (LAC), and femtosecond laser pulses (FLP). High-pressure water jet machining is primarily suitable for rough shaping of components, but it tends to introduce thickness-directional gradients in the machined parts [14] and cannot meet the precision requirements for micro hole manufacturing. Ultrasonic machining, while applicable for complex 3D surface processing, is constrained by vibration amplitude, making it unsuitable for high-precision micro hole fabrication [15]. Laser processing, as a non-contact method, reduces contact stress-induced ceramic damage and enables high-efficiency and high-precision machining [16]; its ease of guidance and focusing also facilitates 3D and special surface processing, rendering it well-suited for ceramic materials. However, laser machining faces challenges in controlling material removal and often results in poor micro-pore formation quality on ceramic matrix composites due to thermal diffusion effects [17]. LAC achieves thermal softening of materials through laser preheating, demonstrating potential in C/SiC machining: Li et al. [18] reported significantly reduced cutting forces and tool wear compared to conventional machining. However, it requires high laser energy, which readily forms heat-affected zones and causes thermal damage. The process is sensitive to parameters, faces challenges in chip evacuation, and carries higher costs. FLP mitigates machining challenges posed by C/SiC’s high hardness and brittleness, reduces tool wear, and prevents traditional defects like chipping and fiber pulling. Its ultra-short pulse mode also eliminates heat-affected zones [19]. Nevertheless, FLP suffers from low material removal rates, chip deposition, and high equipment costs, limiting its industrial application in high-volume micro hole machining.

EDM, characterized by non-contact between the tool electrode and workpiece, avoids the drawbacks of traditional mechanical machining, such as tool wear and contact-induced damage. However, its relatively low processing efficiency has long been a limiting factor, which can be partially mitigated by integrating ultrasonic vibration. In recent years, researchers have begun exploring EDM for ceramic matrix composites: Zhang et al. [20] machined C/SiC using electrodes of varying sizes, demonstrating that micro-EDM is feasible for C/SiC with dimensional accuracy within 0.5 mm; Yue et al. [21] investigated the discharge and erosion behavior of C_f_/SiC during micro-EDM, highlighting the critical role of thermal stress in efficient material removal; He et al. [22] adopted orthogonal experiments to analyze the effects of machining parameters and fiber orientation on wire EDM performance (machining speed and surface roughness) for C/SiC; Xing et al. [23] explored the influence of process parameters (electrode voltage, peak current, pulse width) on material removal rate via orthogonal tests, identifying optimal parameters for maximum efficiency; Liew et al. [24] employed a probe-type vibrator to apply ultrasonic vibrations to a medium fluid during deep micro-porous discharge machining of ceramic materials. Their findings demonstrated that ultrasonic vibrations not only induce agitation but also generate cloud cavitation effects, which facilitate chip removal; Heinz et al. [25] investigated EDM under magnetic field assistance, which improved surface finish, enhanced material removal capability, and promoted process stability; Hocheng H et al. [26] found that the machining damage, recast layer, and mass transfer during EDM processing of C/SiC composites are proportional to the input power.

Despite these preliminary efforts, systematic research on EDM of C/SiC remains scarce. The heterogeneous multiphase structure leads to highly complex discharge behaviors and material removal mechanisms, which are yet to be clearly elucidated. Crucially, the correlation between debris morphology and phase-specific removal has not been systematically established. Key issues such as the synergistic removal mechanism of fiber-matrix interfaces, the quantitative relationship between discharge energy distribution and phase-selective erosion, and the optimization of processing efficiency while maintaining surface integrity remain unresolved. Furthermore, existing electrode designs lack targeted solutions for debris removal and taper control in C/SiC EDM. These knowledge gaps severely restrict the practical application of EDM in high-precision, high-quality machining of C/SiC composite material, underscoring the need for in-depth investigation.

To address the aforementioned gaps, this study focuses on three core innovations: (1) systematically investigating the EDM characteristics of C/SiC by integrating machined surface structure observation, electro erosion debris morphology and composition analysis, and high-speed photography of the discharge process, to clarify the multi-mechanism material removal mechanism; (2) revealing the effects of discharge energy and C fiber orientation on material removal rate and surface quality, providing a theoretical basis for parameter optimization; (3) innovatively developing a hollow internal fluid spiral electrode reaming process that combines spiral structure-induced debris agitation and internal fluid flushing, to improve electro erosion debris removal efficiency, reduce machining defects caused by stress concentration, and mitigate micro hole taper. This work aims to provide a reliable technical approach for high-quality micro hole machining of C/SiC composite material.

## 2. Experimental Setup and Materials

### 2.1. Experimental Setup

**Figure 1:**
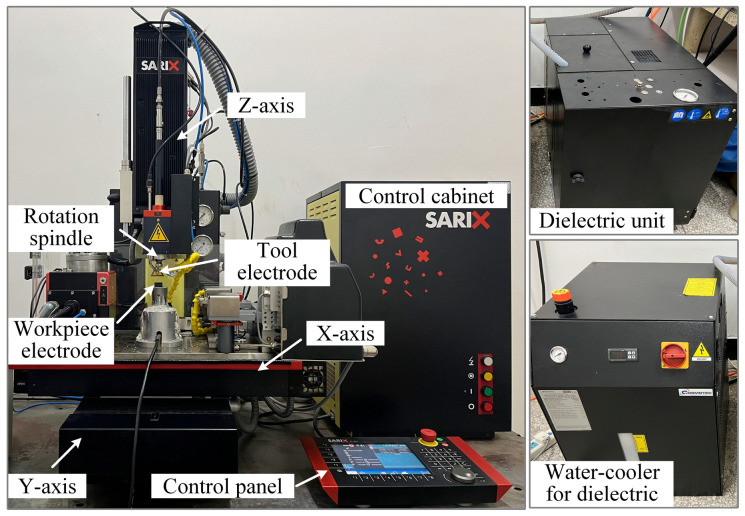
The five-axis coordinated precision EDM machine tools.

The experimental setup employed in this study is a five-axis coordinated precision EDM machine tool (Model: SARIX-100HPM, Sarix SA, Sant’Antonino, Switzerland). The strokes of the X-axis, Y-axis, and Z-axis are 150 mm, 150 mm, and 250 mm, respectively, with a positioning accuracy of ±2 μm and a motion resolution of 0.1 μm. The rotation spindle supports a maximum rotational speed of 600 rpm, enabling electrode rotation to enhance debris removal. All the main functions of the machine are commanded from control panel, where monitoring of machining voltage waveforms and machining time is available. The machine tool and its key components are illustrated in Figure 1.

### 2.2. Materials

**Figure 2:**
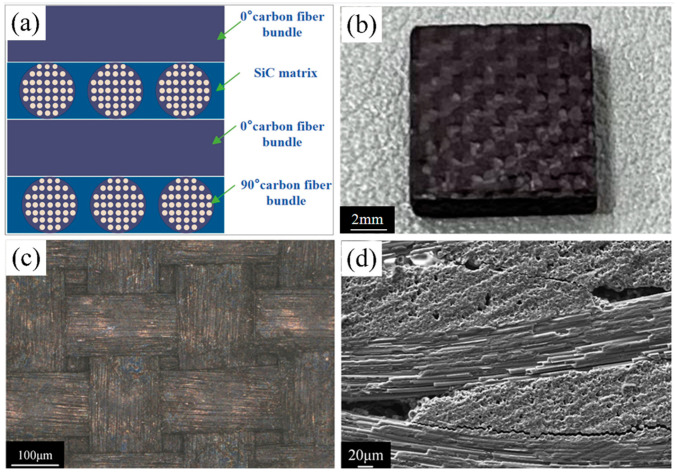
Two-dimensional braided C/SiC composite material. (**a**) Structure schematic diagram. (**b**) Physical picture. (**c**) The surface optical microscope image. (**d**) The cross-sectional SEM image.

C/SiC composite material is a high-performance material composed of SiC matrix and carbon fiber reinforcement. Based on the braiding method, C/SiC composite material is classified into 2D, 2.5D, and 3D types. Compared with 2.5D and 3D braiding, 2D braided C/SiC composite material not only retains the advantages of carbon fiber reinforcement but also simplifies the carbon fiber arrangement to a certain extent, as shown in Figure 2a. The C/SiC composite material blank is cut into 10 mm × 10 mm × 2 mm specimens, which served as the workpiece electrode (Figure 2b). Figure 2c,d show the surface optical microscope image and cross-sectional scanning electron microscope (SEM) image of the C/SiC composite material, respectively. The tool electrode is a tungsten–cobalt alloy rod with a diameter of 0.17 mm in this study.

Due to the addition of carbon fibers, the arrangement of the SiC matrix is altered, and the physical properties of the C/SiC composite material exhibit a range of values due to anisotropy. The physical properties of the C/SiC composite material are listed in Table 1 [27,28,29,30].

In the EDM process, the working fluid is used to remove electro erosion debris generated by discharge, reducing heat generated during machining. The working fluid can also be used to prepare for the next discharge through insulation and deionization effects. Kerosene is selected as the working fluid in this study, and its physical properties are presented in Table 2 [31].

## 3. Results and Analysis

### 3.1. Machining Characteristics of C/SiC Composite Material by EDM

C/SiC composite material is composed of high-strength carbon fibers and brittle silicon carbide ceramic matrix. The multiphase and heterogeneous nature of composite materials makes their EDM mechanism much more complex than that of metal materials, and the removal of materials is the result of multiple mechanisms working together. The EDM characteristics of C/SiC composite material were investigated in this study by observing the machined surface structure, analyzing the morphology and composition of electro erosion debris, and employing high-speed photography to observe EDM process.

**Figure 3:**
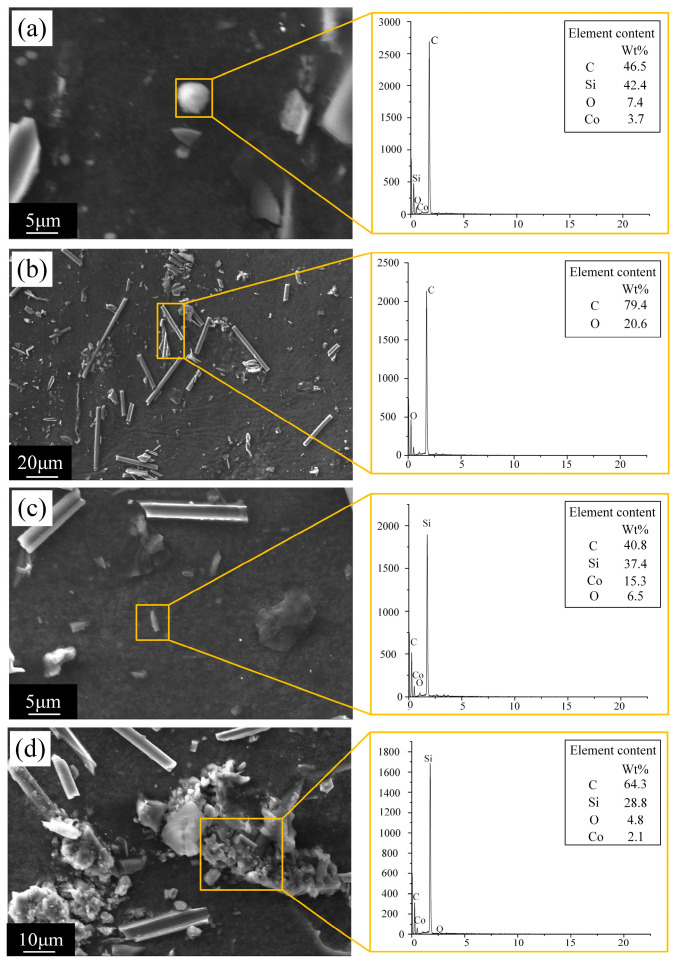
SEM images and EDS spectra of electro erosion debris collected from C/SiC composite material by EDM. (**a**) Spherical debris. (**b**) Cylindrical debris. (**c**) Blocky debris. (**d**) Molten debris.

Figure 3 shows scanning electron microscope (SEM) images and energy dispersive spectrometer (EDS) spectra of electro erosion debris collected from C/SiC composite material by EDM. The electro erosion debris exhibits four primary morphologies: spherical debris, cylindrical debris, blocky debris, and molten debris. For C/SiC composite material, since carbon fibers are good conductors while SiC acts as a semiconductor/insulator, electrical discharge preferentially flows through the conductive carbon fibers. Consequently, the energy within the plasma channel becomes highly concentrated on the carbon fibers within the discharge zone. The carbon fibers are rapidly heated above their vaporization temperature, leading to explosive vaporization. This represents the primary and dominant mechanism for carbon fiber removal, as evidenced by the spherical debris formed in the SEM image (Figure 3a). The EDS spectrum reveals that the surface of the spherical debris primarily consists of four elements: carbon (C), silicon (Si), oxygen (O), and cobalt (Co). The carbon element originates partly from the carbon fiber, while high-temperature decomposition of the kerosene working fluid also generates significant carbon. The silicon element derives from the SiC ceramic matrix. The cobalt element comes from the tool electrode, indicating that high-temperature vaporization also occurs at the tool electrode during EDM, leading to tool electrode wear.

**Figure 4:**
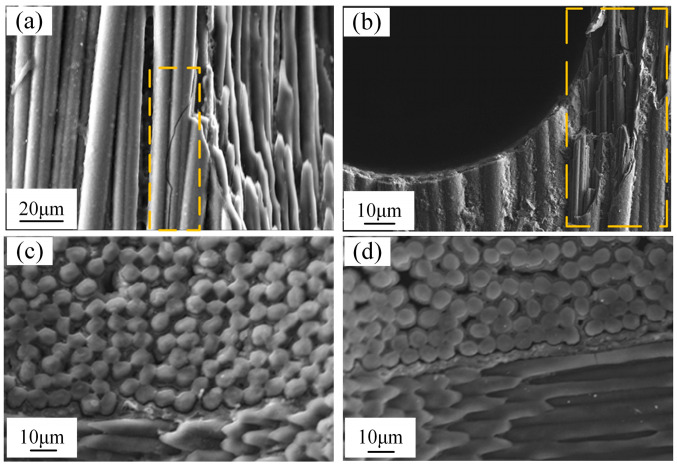
Surface morphology of machined C/SiC composite material by EDM. (**a**) Microcracks on the surface of C fiber. (**b**) Micro damage at hole edge. (**c**) Surface morphology of high discharge energy processing. (**d**) Surface morphology of low-discharge energy processing.

Cylindrical debris was found in the collected electro erosion products of C/SiC composite material machined by EDM, as shown in Figure 3b. If a segment of carbon fiber undergoes severe vaporization or develops microcracks due to thermal stress (Figure 4a), its bond with the SiC matrix will be disrupted. Consequently, the entire fiber deprived of support and constraint will be pulled out or snapped off as a whole under the force of discharge explosions, forming cylindrical debris as illustrated. The edge of the hole will also form micro damage morphology as shown in Figure 4b. Energy dispersive spectroscopy reveals that the debris primarily consists of carbon (C) and oxygen (O). Based on morphology and elemental composition, the cylindrical fragments are identified as carbon fibers. EDS spectra reveal that the debris primarily consists of C and O. Based on morphology and elemental composition, the cylindrical debris is identified as carbon fibers.

Although SiC exhibits poor electrical conductivity, its surface absorbs significant heat during the instantaneous high temperatures of discharge. As SiC is a brittle ceramic, this intense localized heating induces substantial thermal stress, leading to the formation of microcracks in the SiC matrix. As the surrounding carbon fibers are eroded away, the SiC matrix originally reinforced by these fibers loses its structural support. Under the explosive force of the discharge, the SiC matrix undergoes brittle fracture, flaking off in blocky or granular fragments, forming blocky debris, as shown in Figure 3c. Within the high-temperature core region at the discharge point, SiC can be directly sublimated or decomposed into silicon and carbon. Molten debris is also one of the primary forms of electro erosion products, as shown in Figure 3d. Although irregular in shape, it exhibits typical characteristics of melting and recrystallization. Elemental analysis of the molten debris reveals it primarily consists of four elements: C, Si, O, and Co. The results indicate that melting is also an important removal mechanism in the EDM of C/SiC composite material.

Collected debris exhibits four distinct morphologies, each correlating with a specific removal mechanism. Spherical debris (primary carbon fiber product) forms via explosive vaporization from concentrated plasma channel energy, containing C (fibers, kerosene thermal decomposition), Si (SiC matrix), O, and Co (tool electrode). Cylindrical debris (predominantly C/O, fragmented fibers) stems from carbon fiber pull-out/fracture after interfacial debonding from SiC matrix via thermal stress or vaporization. Blocky debris results from brittle fracture and spalling of SiC matrix (devoid of carbon fiber reinforcement) due to intense localized thermal stress-induced microcracks. Molten debris (melting-resolidification features) contains C, Si, O, and Co, confirming melting as another key removal mechanism for C/SiC machined by EDM. Notably, element Co presence across all debris confirms tool electrode wear via high-temperature vaporization.

Figure 4c,d show the surface of the C/SiC composite material after EDM exhibits an uneven pitted structure, with the raised carbon fiber bundles forming a stark contrast against the recessed SiC matrix. This phenomenon arises from the significant differences in thermophysical properties between the carbon fibers and the SiC matrix within the C/SiC composite material, leading to their selective removal. Figure 4c,d also reveal that the degree of surface irregularity varies with different discharge energies. The equipment is equipped with controllable modules for both high and low discharge energies. By switching between different modules, precise regulation of discharge energy can be achieved. Higher discharge energy will result in greater surface irregularities, indicating that higher energy processing removes a larger volume of SiC matrix (Figure 4c). Although the current preferentially passes through carbon fibers and vaporizes them, higher energy pulses generate extremely strong explosive forces and thermal effects, which directly “bombard” the SiC matrix that has become fragile due to the loss of carbon fiber support. The SiC matrix is removed in the form of particle peeling, resulting in a relatively larger volume of removal. When processing with lower discharge energy, the energy of low-energy pulses is very concentrated, and the energy precisely acts on the C fibers at the discharge point, vaporizing them and increasing the removal ratio of carbon fibers (Figure 4d). For SiC matrix, due to insufficient explosive force, it is difficult to cause large-scale peeling, mainly relying on thermal stress to generate microcracks or material sublimation. At this point, the total amount of material removed by a single pulse is very small, with a higher proportion of vaporized C fibers.

**Figure 5:**
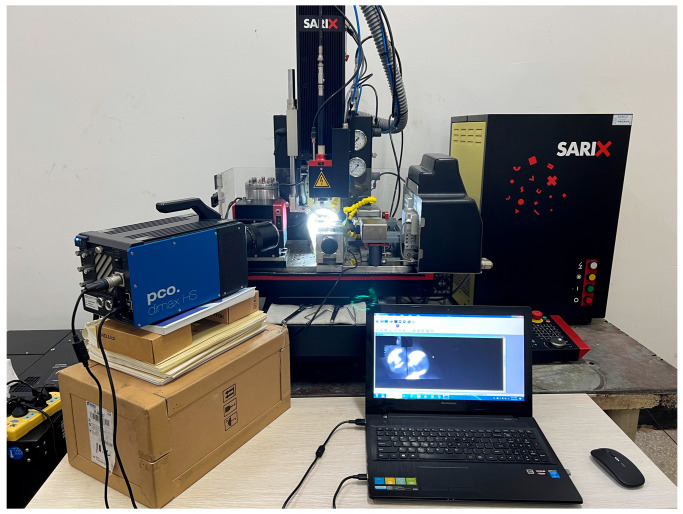
Processing process captured by high-speed camera.

**Figure 6:**
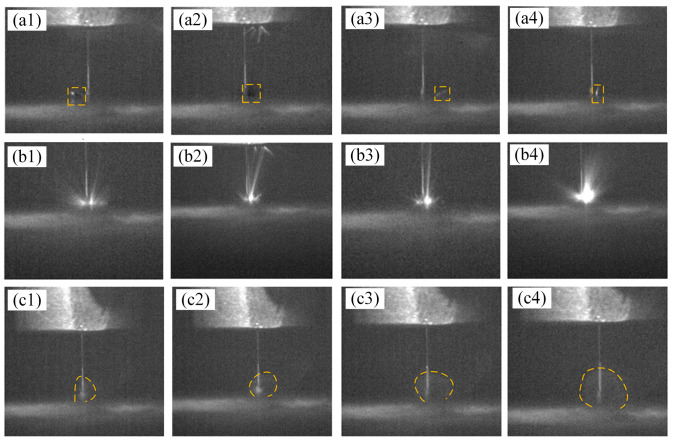
Machining process images captured by high-speed camera. (**a1**–**a4**) Fracturing and splattering of C fibers. (**b1**–**b4**) Spark discharge during the machining process. (**c1**–**c4**) Black smoke observed during the machining process.

To further investigate the characteristics of C/SiC composite material by EDM, a high-speed camera was employed to observe the discharge zone during EDM (Figure 5). The high-speed camera captured the instantaneous fracturing and spattering of C fibers, as shown in Figure 6(a1–a4). The black particles represent these fractured C fiber fragments, which are ejected in large quantities at the start of EDM. When the discharge channel is formed instantaneously during EDM, the outermost layer of carbon fibers and the SiC matrix on the workpiece surface undergo drastic changes due to high temperature, generating significant thermal stress. With the accumulation of thermal stress, the ultimate limit that carbon fibers can withstand is reached, and the surface carbon fibers instantly break, with fragments irregularly scattered in various directions under the force of discharge explosions, forming cylindrical debris. This phenomenon is characteristic of micro-EDM processing of C/SiC composite material.

As the tool electrode continues to move downward and perform discharge machining, the surface carbon fiber layer and SiC matrix are gradually eroded away, revealing the denser carbon fiber bundle structure. As the reinforcing phase in C/SiC composite material, the carbon fiber bundle exhibits significantly higher conductivity than the surface carbon fiber layer and SiC matrix. It clearly demonstrates in Figure 6(b1–b4) that as the machining process continues, the spark discharge becomes increasingly intense. Additionally, carbon fiber readily undergoes combustion reactions with oxygen at high temperature, producing CO or CO_2_ gases, while the high temperatures generated by electrical discharge provide energy for the oxidation reaction. The oxidation combustion of carbon fiber is also one of the material ablation processes. This is a highly exothermic reaction that accelerates material removal.

(1) C + O_2_ → CO_2_

(2) 2C + O_2_ → 2CO

During the EDM process of C/SiC composite material, the generation of black smoke was also observed, as shown in Figure 6(c1–c4). SiC matrix also decomposes and vaporizes at high temperature (Formula 3), the decomposition of SiC directly produces free C, which further increases the C particle content in black smoke. Decomposition products also include Si vapor. Vaporized Si condenses into blocky silicon particles upon cooling or reacts with O_2_ in the air to form SiO_2_ particles (Formula 4), becoming a part of the black smoke. Since kerosene is used as the working fluid in the EDM process, it undergoes thermal cracking at high discharge temperature, thereby generating carbon smoke. In summary, the black smoke produced during the EDM of C/SiC composite material primarily consists of CO and CO_2_ gases generated by the combustion reaction of carbon fibers with O_2_ at high temperature, free C and Si particles resulting from SiC decomposition, and carbon smoke produced by the thermal cracking of kerosene.

(3) SiC (s) → Si (g) + C (s)

(4) SiC + 2O_2_ → SiO_2_ + CO_2_

### 3.2. Effect of Carbon Fiber Orientation Angle on Material Removal Process

**Figure 7:**
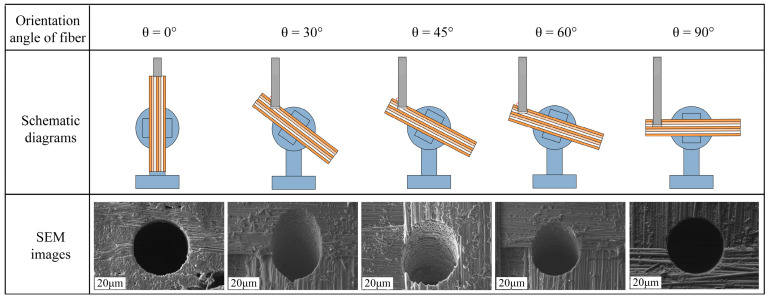
The schematic diagrams and hole SEM images of different fiber orientation angles.

**Figure 8:**
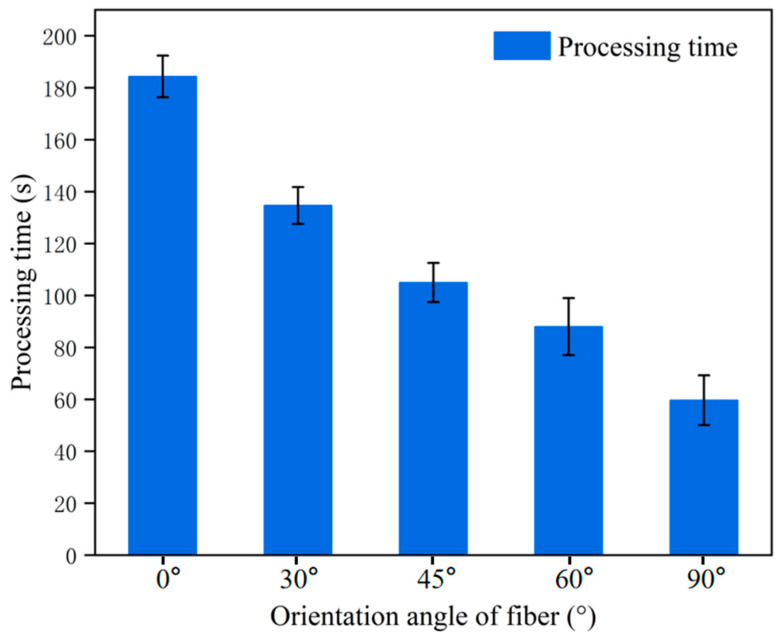
Processing time of different C fiber orientation angles.

C fiber is a good conductor, while the SiC matrix is a semiconductor or insulator; the flow path of the current within the material strongly depends on the orientation and distribution of the fibers. The melting points, vaporization points, and thermal conductivities of C fibers and SiC differ significantly; heat conduction and accumulation also vary depending on fiber orientation. Therefore, this paper investigates the influence of the C fiber orientation angle on the EDM process of C/SiC composite material. The fiber orientation angle θ is defined as follows: a fiber orientation angle of 0° is parallel to the tool electrode feed direction, while a fiber orientation angle of 90° is perpendicular to the tool electrode feed direction. The schematic diagrams and hole SEM images of different fiber orientation angles are shown in Figure 7. The influence of different fiber orientation angles on material removal rate was also investigated. The time required to remove the same volume of material was used as the evaluation method for material removal rate. The processing time under different C fiber orientation angles θ is shown in Figure 8. When the C fiber orientation angle θ = 90°, the shortest processing time was achieved. As the C fiber orientation angle θ gradually decreased, the processing time exhibited an increasing trend. When the C fiber orientation angle θ = 0°, the longest processing time was recorded.

When the fiber is perpendicular to the tool electrode feed direction (θ = 90°), the discharge point will directly act on the cross-section of the fiber. The carbon fiber undergoes intense vaporization or develops microcracks due to thermal stress, disrupting its bond with the SiC matrix. Consequently, the entire fiber is either pulled out or snapped under the combined force of the discharge explosion and the erosion force of the working fluid, rather than being completely ablated and melted. This approach proves more efficient than relying solely on high-temperature melting/vaporization of its cross-section. When fibers are fed parallel to the tool electrode (θ = 0°), discharge occurs along the fiber’s axial direction. Since C fibers exhibit high axial strength and excellent thermal conductivity, melting or vaporizing them requires highly concentrated and powerful discharge energy, resulting in relatively lower material removal rate and the longest processing time.

### 3.3. Micro Hole Quality by EDM

**Figure 9:**
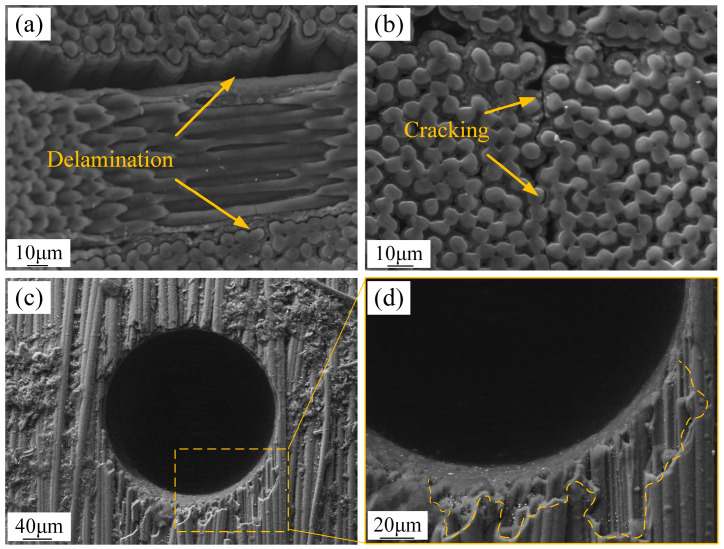
Hole defects of C/SiC composite material processed by EDM. (**a**) Delamination between alternating layers of 0° and 90° fiber bundles. (**b**) Cracking at the interface between SiC matrix and C fibers. (**c**,**d**) Fiber delamination fractures at the hole entrance.

During EDM processing of C/SiC composite material, defects such as the so-called delamination phenomenon can be observed, as illustrated in Figure 9. Delamination occurs between alternating layers of 0° and 90° fiber bundles (Figure 9a), while cracking also appears at the interface between SiC matrix and C fibers (Figure 9b). Notably, fiber delamination fractures also occur at the hole entrance (Figure 9c,d), posing a significant issue for hole formation quality that cannot be overlooked.

Two-dimensional C/SiC composite material is the most commonly preformed structure, featuring woven fiber arrangements between layers but lacking fibers oriented along the Z-axis. Compared to 2.5D and 3D C/SiC composite materials, 2D C/SiC composite material exhibits weaker bonding along the Z-axis. Localized thermal stresses generated during EDM readily overcome interlaminar bonding strength, leading to delamination defects. Differences in thermal conductivity, melting point, and boiling point between C fibers and the SiC matrix cause the material removal rate to vary along the fiber orientation, this leads to cracks and delamination between C fibers and the SiC matrix.

**Figure 10:**
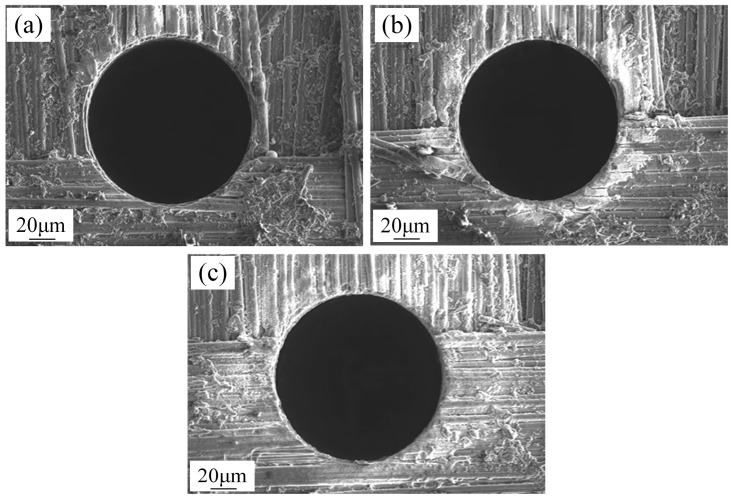
The ejected debris zone under different peak currents. (**a**) 20 A, (**b**) 30 A, (**c**) 40 A.

Under identical process parameters, increasing peak current progressively enlarges both the ejected debris zone and the heat-affected zone, as shown in Figure 10. On one hand, increased peak current enhances discharge energy, boosting material removal per unit time and expanding the heat-affected zone. On the other hand, it also increases the explosive force during EDM, prolonging the ejection distance of electro erosion debris—a phenomenon consistent with general principles observed in EDM of metallic materials.

### 3.4. Hollow Internal Flushing Helical Tool Electrode Reaming Process

**Figure 11:**
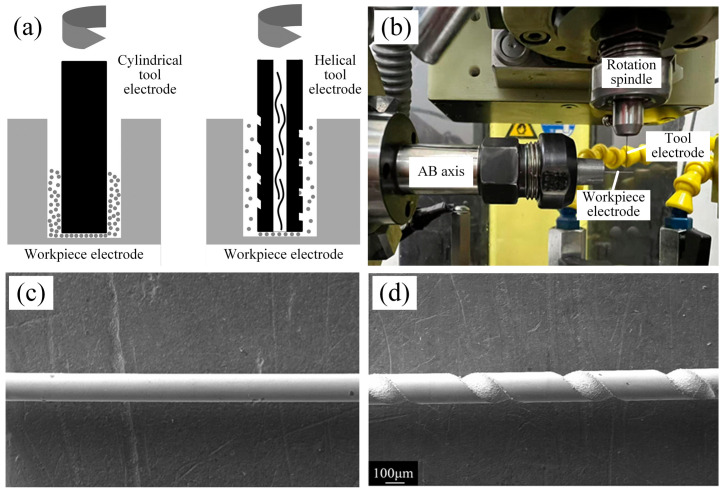
Hollow internal flushing helical tool electrode. (**a**) Removal principal diagram of electro erosion debris. (**b**) Preparation method for the groove of the helical electrode. (**c**) Cylindrical tool electrode. (**d**) Helical tool electrode.

In order to improve the removal efficiency of electro erosion debris, reduce machining defects caused by stress concentration, and enhance the quality of micro hole in C/SiC composite material by EDM, a hollow internal flushing helical tool electrode was developed in this paper, as shown in Figure 11. This design suppresses micro hole defects by improving debris removal efficiency and optimizing the flow state of the working fluid (Figure 11a). Compared to cylindrical tool electrode, the grooves of the helical electrode can both accommodate and remove debris. Furthermore, the high-speed rotation of the spindle during machining generates a pressure gradient in the gap flow field. This expels chips upwards along the helical grooves, thereby improving debris removal efficiency. The hollow tool electrode directly injects working fluid into the discharge zone through its internal channels, creating directed fluid flushing that rapidly expels electro erosion debris from the narrow discharge gap. Figure 11b illustrates the preparation method for the groove of the helical electrode, achieved by electrical discharge milling. Figure 11c,d show a comparison of SEM images between cylindrical electrode and prepared helical electrode.

**Figure 12:**
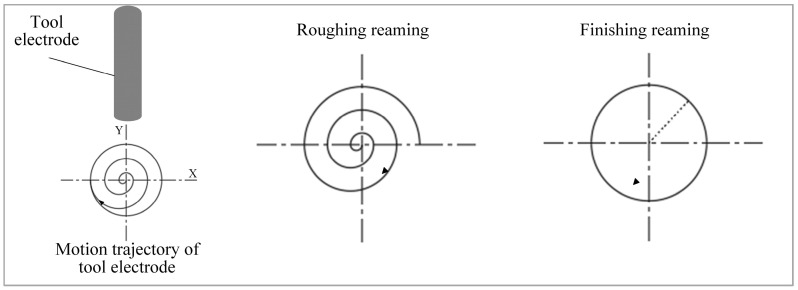
The electrode’s motion trajectory during reaming.

A hollow internal fluid spiral electrode reaming process has been developed to enhance micro hole forming quality. Reaming utilizes discharge along the side surface of tool electrode to enlarge pre-machined micro hole. The reaming process comprises roughing and finishing operations. Roughing removes machining residues from the inner wall, while finishing further improves micro hole formation quality by reducing taper. The electrode’s motion trajectory during reaming is illustrated in Figure 12.

**Figure 13:**
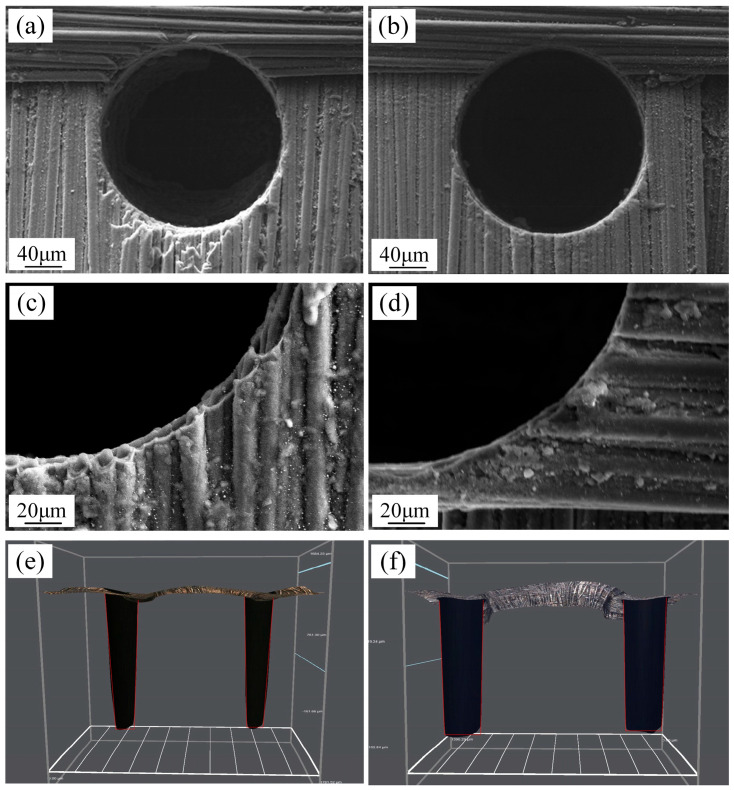
The micro hole morphology before and after the reaming process of blind holes. (**a**,**c**) Micro hole edge morphology before reaming. (**b**,**d**) Micro hole edge morphology after reaming. (**e**) Three-dimensional structural diagram of micro hole before reaming. (**f**) Three-dimensional structural diagram of micro hole after reaming.

The micro hole morphology before and after the reaming process is shown in Figure 13. Figure 13a,c reveal a distinct delamination phenomenon at the micro hole entrance edge before reaming. Figure 13b,d demonstrate a significant reduction in this delamination effect after reaming. The three-dimensional micro-porous images in Figure 13e,f further indicate that the reaming process reduces micro hole taper and enhances the formation quality of the micro hole.

## 4. Conclusions

This paper focuses on addressing the key challenge of poor micro hole forming quality in EDM of C/SiC composite material, systematically investigating the EDM characteristics of C/SiC composite material, and innovatively developing a hollow internal fluid spiral electrode reaming process for micro hole machining. The specific conclusions are as follows:

(1) The debris collected during the EDM process of C/SiC composite material exhibits four distinct morphologies: spherical, cylindrical, blocky, and molten debris. This indicates that the process of C/SiC composite material by EDM includes the melting and explosive vaporization of the material. There is also overall pull-out and fracture of C fibers, as well as brittle fracture and spalling of SiC matrix. These findings clarify the material removal mechanism of C/SiC composite material in EDM with the hollow internal fluid spiral electrode.
(2) The degree of surface irregularity varies with different discharge energies. Higher discharge energy will result in greater surface irregularities, indicating that higher energy processing removes a larger volume of SiC matrix. The energy of low-energy pulses is very concentrated, and the energy precisely acts on the C fibers at the discharge point, with a higher proportion of vaporized C fibers. This conclusion enables targeted regulation of discharge energy for the hollow internal fluid spiral electrode reaming process, balancing machining efficiency and surface quality.
(3) Different fiber orientation angles have a great influence on material removal rate. When the C fiber orientation angle θ = 90°, the shortest processing time was achieved. As the C fiber orientation angle θ gradually decreased, the processing time exhibited an increasing trend. When the C fiber orientation angle θ = 0°, the longest processing time was recorded. This finding provides a parameter adaptation basis for the hollow internal fluid spiral electrode reaming process to handle C/SiC composite material with different fiber orientations, expanding the process’s application scope.
(4) To address the technical challenges encountered in traditional EDM of C/SiC composites, such as poor chip removal, there are frequent abnormal discharges and excessive taper in micro holes. A hollow internal fluid spiral electrode reaming process. Compared to traditional methods, the hollow internal flushing helical tool electrode can greatly improve the efficiency of debris removal and reduce the occurrence of abnormal discharge phenomena. The reaming process can reduce the micro hole taper and enhance the formation quality of the micro hole. This process provides a new technical approach for high-quality micro hole machining of C/SiC composite material, which is the core novelty of this research.

## Figures and Tables

**Table 1:** Physical properties of the C/SiC composite material.

Physical Properties	Value
Density (kg/m^3^)	2100
Melting point (°C)	2730
Specific heat capacity (J/g·°C)	0.7–1.2
Thermal conductivity (W/m·K)	5–6.3

**Table 2:** Physical properties of kerosene.

Dielectric Constant	Insulating Strength (MV/m)	Flash Point (°C)	Density (kg/m^3^)	Ignition Point (°C)
3	14.22	134	813	243

## Data Availability

The raw data supporting the conclusions of this article will be made available by the authors on request.

## Abbreviations

The following abbreviations are used in this manuscript:

EDM	Electric discharge machining
SEM	Scanning electron microscope
EDS	Energy dispersive spectrometer
LAC	Laser-assisted cutting
FLP	Femtosecond laser pulses
